# Validation of the Upper Limb Functional Index on Breast Cancer Survivor

**DOI:** 10.3390/ijerph20064997

**Published:** 2023-03-12

**Authors:** Jaime Martín-Martín, Bella Pajares-Hachero, Emilio Alba-Conejo, Nuria Ribelles, Antonio I. Cuesta-Vargas, Cristina Roldán-Jiménez

**Affiliations:** 1 Legal and Forensic Medicine Area, Department of Human Anatomy, Legal Medicine and History of Science, Faculty of Medicine, University of Malaga, 29010 Málaga, Spain; 2 Grupo Clinimetría en Fisioterapia (F-14), Instituto de Investigación Biomédica de Málaga (IBIMA), 29590 Málaga, Spain; 3 UGCI Oncología Médica, Hospitales Universitarios Regional y Virgen de la Victoria, 29010 Málaga, Spain; 4 Instituto de Investigación Biomédica de Málaga (IBIMA), 29590 Málaga, Spain; 5 Department Physiotherapy, Faculty of Health Sciences, University of Malaga, 29071 Malaga, Spain; 6 School of Clinical Science, Faculty of Health, Queensland University of Technology, Brisbane, QLD 4000, Australia

**Keywords:** upper extremity, breast cancer, psychometrics, patient-reported outcome, functional assessment

## Abstract

Breast cancer survivors (BCS) may face functional alterations after surgical intervention. Upper Limb Disorders (ULDs) are highly prevalent even years after a diagnosis. Clinicians may assess the upper limbs after breast cancer. The Upper Limb Functional Index (ULFI) has been validated across different populations and languages. This study aimed to assess the psychometric properties of the Upper Limb Functional Index Spanish version (ULFI-Sp) in the BCS. Methods: A psychometric validation study of the ULFI-Sp was conducted on 216 voluntary breast cancer survivors. The psychometric properties were as follows: analysis of the factor structure by maximum likelihood extraction (MLE), internal consistency, and construct validity by confirmatory factor analysis (CFA). Results: The factor structure was one-dimensional. ULFI-Sp showed a high internal consistency for the total score (α = 0.916) and the regression score obtained from MLE (α = 0.996). CFA revealed a poor fit, and a new 14-item model (short version) was further tested. The developed short version of the ULFI-SP is preferable to assess upper limb function in Spanish BCS. Conclusions: Given the high prevalence of ULD in this population and the broader versions of ULFI across different languages, this study’s results may be transferred to clinical practice and integrated as part of upper limb assessment after breast cancer.

## 1. Introduction

Breast cancer is one of the most prevalent cancers worldwide in women [1]. After surgical treatment, breast cancer survivors (BCS) commonly face functional alterations in the upper limbs, which may persist even after surgery [2]. Specifically, more than 50% of BCS present upper limb dysfunction (ULD) 6 years post-diagnosis [3]. The most common ULD types are pectoralis tightness, lymphedema, and higher rates of rotator cuff disease [4]. Other limitations for women treated with breast cancer surgery are a strength deficit, the restriction of range movement, pain, a frozen shoulder, and axillary web syndrome [4,5,6].

Clinicians commonly use patient-reported outcomes (PROs) to assess a patient’s symptoms or functional status [7,8]. These subjective data can help clinicians better understand how a condition or disease influences a patient’s capabilities, functioning, and symptoms [9]. There are many validated PROs to measure the functionality of the shoulder and upper limbs [10,11,12,13,14]. Among them, the Upper Limb Functional Index (ULFI) was developed to address the limitations of previous PROs, showing superior practical characteristics, clinical utility, and comparable psychometric properties [14]. Items from the ULFI were generated after a reduction of 850 item statements from 125 PROM questionnaires. Selected items are consistent with the definition of activity limitation defined by the World Health Organization’s International Classification of Impairments, Activities, and Participation [14]. They evaluate the patient’s difficulty carrying out activities due to alterations in the upper limb. The ULFI consists of twenty-five related questions with a three-point response option (no/sometimes/yes) [15]. The answers are added directly and become a percentage based on 100. Its practical characteristics, such as no missing response or a combined patient completion and therapist scoring time lower than 3 min, contribute to improved efficiency in the clinical and therapeutic settings [14]. Additionally, these one-dimensional PROs have shown excellent reliability, internal consistency, concurrent validity, and responsiveness, among other psychometric properties [15]

In the breast cancer population, there are PROs that are used to assess the upper limbs [16], including those related to surgical intervention [17] or measuring the impact of arm morbidity on the quality of life [18]. There are three specific questionnaires designed for the evaluation of the upper limbs in breast cancer survivors: Kwan’s Arm Problem Scale (KAPS) [19], Wingate [20], and the Upper Limb Disability Questionnaire (ULDP) [21]. However, only KAPS has demonstrated its psychometric properties [19]. In contrast, some PROs were not designed for BCS but were validated later in this population, such as the QUICK-Dash [22], Upper Extremity Functional Index (UEFI) [23], Oxford Shoulder Score (OSS), and SPADI [24]. Only OOS and SPADI have been validated in Spanish in this population [24].

The Spanish version of the ULFI has been used to analyze factors associated with the upper limb function [25]. Given its clinical utility, the Spanish version of the ULFI has been used to assess therapeutic exercise programs in a real-world setting [26,27] and to measure changes after an exercise intervention in metastatic breast cancer patients [28]. However, the ULFI psychometric properties have not been analyzed in the breast cancer population. Therefore, this study aimed to evaluate the critical psychometric properties of the ULFI in BCS.

## 2. Materials and Methods

### 2.1. Design

This cross-sectional study recruited a population of female BCS to evaluate the psychometric properties of ULFI in terms of structural validity, reliability, and factor analysis.

Clinical data were collected on the years since diagnosis, type of surgical intervention (breast-conserving or mastectomy), type of adjuvant treatment (radiotherapy, chemotherapy, hormone therapy, or monoclonal antibody), and current treatment (none, radiotherapy, monoclonal antibody, or hormone therapy).

The Spanish version of the Upper Limb Functional Index (ULFI) has excellent psychometric properties for reliability and validity [29]. The questionnaire consists of 25 questions that are transferable to a 100-point scale, in which a higher percentage implies less functionality. Results were subtracted to 100 to be expressed as a percentage of functionality (%). All study participants completed the questionnaire.

### 2.2. Participants, Setting, and Procedure

A total of 216 voluntary BCS from Virgen de la Victoria University Hospital of Malaga participated in the study. Medical oncologists recruited women from the Medical Oncology Unit at the hospital. Informed consent from the participants was obtained for the present study. Subjects were asked to fulfil the questionnaire as part of an assessment. A physiotherapist (CRJ) supervised the procedure. The inclusion criteria were BCS who had been surgically treated for their primary tumor with no evidence of recurrence at the time of recruitment. The exclusion criteria were under 18 years old and low reading comprehension due to completing the questionnaire.

### 2.3. Statistics

A descriptive statistic of the participants was made with a mean and standard deviation of the demographic variables. The Kolmogorov–Smirnov test by one sample (significance > 0.05) was used to calculate the sample’s distribution and normality.

#### 2.3.1. Structural Validity

The chi-square test was used to show differences between observed covariance and expected matrices. A Kayser–Meyer–Olkin (KMO) Measure of Sampling Adequacy (>0.70) [30] and Bartlett’s Test of Sphericity (*p* < 0.05) [31] were calculated to evaluate the suitability of the ULFI data for factor analysis.

Construct validity and factor structure were determined through the use of Maximum Likelihood Extraction (MLE), with the requirements for extraction being the satisfaction of all three points: scree plot inflexion point, Eigenvalue > 1.0, and accounting for > 10% of the variance [32]. The recommended minimum ratio of five participants per item was satisfied [32]. According to Costello and Osborne, a cutoff point of 0.3 item loading was considered the minimum load per item [32]. In this way, Hair et al. also indicated that factor loadings above 0.3 may be considered adequate; significant 0.4 loadings and factor loadings greater than 0.5 would be considered practically significant [33].

#### 2.3.2. Reliability: Internal Consistency

ULFI internal consistency was evaluated by Cronbach’s α coefficients calculated at an anticipated value range of 0.80–0.95 [34,35]. A regression score obtained from factorial extraction by MLE was used for reliability between each item and ULFI total score. Ranges were expressed by Intraclass Correlation Coefficient (ICC 95%).

#### 2.3.3. Factor Analysis

Correlation between items and confirmatory factor analysis (CFA) was made to test whether measures of a construct are consistent with this construct’s nature. A comparative fit index (CFI) was performed to measure the relative improvements in adjusting the study model against the original model. An index close to 0.95 was considered acceptable, while an index of 0.97 was considered indicative of a good fit [36,37]. Root mean square error of approximation (RMSEA) was used to avoid sample size problems when analyzing the discrepancy between the hypothesized model with the optimally chosen parameter estimates and the population’s covariance matrix. RMSEA with 90% confidence intervals was analyzed (RMSEA ≤ 0.08 indicates an acceptable fit, and ≤ 0.05 indicates a good fit [36]).

Factor structure by MLE was carried out with the software Statistical Package Social Science Version 25.0 (SPSS 25.0) [38] for Windows. AMOS was employed for CFA [39].

## 3. Results

### 3.1. Sample Description

BCS were aged 51.64 (9.10). The mean values and standard deviation in the ULFI-Sp questionnaire in breast cancer survivors were 71.66 ± 21.57%. Descriptive and anthropometric variables are shown in Table 1.

### 3.2. Structural Validity

**Figure 1:**
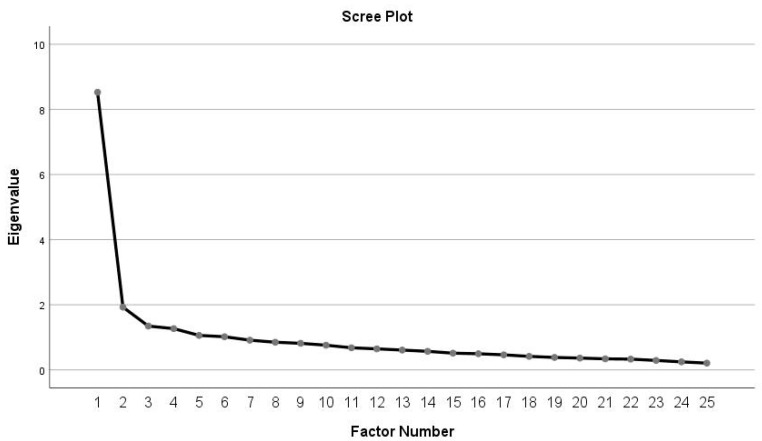
Scree plot of the exploratory one-factor solution.

The Kaiser–Meyer–Oklin test determined the correlation matrix (0.889) for the ULFI-Sp in female BCS and the Bartlett’s Test of Sphericity (chi-squared value = 2087.167 and df 300, *p* < 0.000). These values indicate that there is little likelihood of it being an identity matrix, meaning it was appropriate to perform the EFA analysis (see Figure 1). An MLE factor analysis identified six factors with an eigenvalue greater than one, explaining 34.10%, 7.70%, 5.37%, 5.06%, 4.42%, and 4.06% of the variance, respectively, which represents 60.54% of the variance explained. Therefore, only one factor accounted for > 10% of the variance. The other five factors were shown to be after the scree plot initial inflexion point and were not extracted (Figure 1).

The ULFI-Sp showed a high degree consistency, as illustrated by the high Cronbach value (α = 0.916) with an individual item range between 0.868 and 0.875. The correlation between the ULFI total score and the regression score obtained from the one-factor solution MLE was also high (α = 0.996). The correlation between items and the regression score from MLE, communalities, and the average score for each item is shown in Table 2. The results of the CFA revealed a poor fit, with CFI = 0.802 and RMSEA = 0.075 (Table 3).

Therefore, a new 14-item model (short version) was tested with those items with communalities higher than 0.3. According to Costello and Osborne, a cutoff point of 0.3 item loading was considered the minimum load per item [32].

### 3.3. New 14-Item Model

The chi-square test was significant for the new tested model: χ^2^ = 1171.591 *p* < 0.000. Moreover, the rest of the fit indicators suggested that the model fit the data well, with the adequate RMSEA (RMSEA = 0.069; 90%CI 0.056–0.082) and CFI with the fit (CFI = 0.905). Table 2 shows items from the 14-item model with standardized factor loadings. The psychometric properties of both models are summarized in Table 3.

## 4. Discussion

In this study, the psychometric properties of the ULFI-Sp were analyzed in a sample of BCS, resulting in a new ULFI-Sp short version. Psychometric properties were evaluated following Costello and Osborne [32]. ULFI-Sp showed a high internal consistency, and the KMO showed that the ULFI was suitable for the EFA analysis, resulting in a one-factor solution. However, a factor analysis by CFA revealed a poor fit, and a new 14-item model was tested.

Although previous PROs have been validated in BCS [24], this study offers the psychometric properties of the ULFI and the development of a new short version in a wide sample of 216 BCS. A factor analysis by CFA was also tested.

### 4.1. Structural Validity

In the present study, the KMO showed that the ULFI was suitable for an EFA analysis. The one-factor solution that emerged in the factor analysis accounted for 60.54% of the total variance. However, CFAs factor analysis revealed a poor fit, and a new 14-item model was tested from those items with higher communalities (Table 2). The unidimensional structure of the ULFI-Sp tested in BCS should be noted, as this population suffers from additional symptoms in addition to ULD [40]. The original version of the ULFI in English has one dimension factor [15], and its analysis showed six factors with eigenvalues > 1.0, like what was found in the present study. In addition, authors from the original version found that 14 items scored below 0.50, suggesting that the questionnaire could be shortened in future studies [15]. The Italian version of the ULFI with 19 items also found a one-factor solution, which supports the construct validity of the questionnaire [41]. A unidimensional structure is vital to accurately reflect the measured region with a single summated score [42]. Other ULFI versions, such as Turkish [43] or Urdu [44], have shown a two-factor structure in patients with musculoskeletal disorders. Bidimensional questionnaires such as DASH have been reduced to a one-factor structure by eliminating item redundancy [42]. However, this nine-item shortened version has only been validated in patients suffering from different upper limb musculoskeletal conditions [42].

### 4.2. Reliability: Internal Consistency

The original version of the ULFI-Sp has a high internal consistency (α = 0.94) validated in patients with variable alterations of the upper limb [29]. Internal consistency of OSS-Sp was (α = 0.94) and for the SPADI-Sp pain subscale (α = 0.93) and disability subscale (α = 0.95), these values represent a total internal consistency of (α = 0.96) [24]. The KAPS also demonstrated a high internal consistency of (α = 0.94) [19]. In this study, ULFI-Sp showed good internal consistency (α = 0.916) for the total score and α = 0.996 for the regression score obtained from MLE. Likewise, the reliability and validity of the UEFI were demonstrated in BCS after surgery. The test–retest results obtained were 0.87, and the UEFI correlation compared to QuickDASH in the same population was 0.79 [23].

Similarly, reduced versions of DASH (30 items), QuickDASH (11 items), and QuickDASH of 9 items (QuickDASH-9) have been developed in patients with upper limb musculoskeletal conditions [42]. The nine-item version has a good internal consistency (α = 0.93), as well as high levels of correlation with both the original DASH version (r = 0.97) and QuickDash (r = 0.99). Similarly, the correlation of the QuickDash-9 against the original ULFI was good (r = 0.85). This smaller version means a lower loss in response rates [42].

### 4.3. Factor Analysis

One of the strengths of the present study was that factor analysis by CFA was tested. Both ULFI-Sp models had RMSEA values ≤ 0.08, indicating acceptable fit [36]. Although none of the models tested reached CFI values for acceptable fit [36,37], CFI values from the short version showed a better adjustment of the study model (Table 3). Therefore, although ULFI-Sp presented good psychometric properties in terms of structural validity and internal consistency, the short version’s use is preferable when contemplating factor analysis.

The SPADI questionnaire of 13 items was also reduced and translated into Spanish with ten items [45]. Validation was performed in upper limb musculoskeletal disorders, showing comparative fit index (CFI 0.98), normed fit index (NFI 0.95), goodness of fit index (GFI 0.95), RMSEA 0.06 (90% CI 0.04 to 0.09), and good internal consistency (α = 0.90) [45].

### 4.4. Short Version

A QuickDASH version of 11 items was validated in BCS. The questionnaire reliability was high (α = 0.93). The intra-class coefficient was 0.78. A factor analysis showed one factor with an Eigenvalue of 6.7; this factor explained 61% of the variance. [22]. In the analysis of the goodness of fit of the ULFI-Sp in BCS (Table 3), the CFI and RMSEA values of the proposed model (14 items) are better than those of the original model (25 items). The CFI and RMSEA values have a reasonable adjustment between the proposed and the original model.

Although there are several short versions of questionnaires to measure upper limb function in the literature [15,42,45], it should be noted that the short version developed in the present study emerged from specific data from a BCS. In this regard, several items related to BCS symptoms are kept in the short version (see Table 3). Firstly, items 3 and 21 refer to carrying and moving heavy loads. It is well known that patients suffer from a loss of strength after breast surgical intervention [46]. Furthermore, BCS may also face kinesiophobia [47,48] and pain catastrophism [49], which may negatively influence the capacity for lifting heavy weights with the affected arm. Secondly, item 19 refers to the capability to lift things at or above the shoulder. There is an explicit limitation in shoulder ROM [46,50]. More specifically, patients suffering from affectations, such as West syndrome [5] will feel this function impaired. Finally, items 22 and 24 refer to dropping things and tasks related to fine motor skills, which may be impaired in the presence of peripheral nervous system disorders [51].

### 4.5. Clinical Implications

Other questionnaires, such as SPADI, have been integrated as part of an early warning surveillance system to detect ULD in BCS [52]. Given the broader versions of the ULFI across different languages [29,43,53,54], the ULFI may be integrated as part of the upper limb assessment after breast cancer. There is a high prevalence of patients with these characteristics [1], and the questionnaires have proved helpful for clinicians in assessing patients’ capabilities [9]. The application of the ULFI in this population (which, even after the operation, has limitations in the functionality of the upper limb [2,3]) could have a high impact and the possibility of transferring results to clinical practice.

### 4.6. Limitations

The present study has several limitations. The psychometric analysis of the ULFI-Sp did not include responsiveness, so the test–retest reliability can be compared to other questionnaires. Furthermore, as a new short version was developed, future studies should also address the correlation between the ULFI-Sp short version and different reduced versions of questionnaires, such as the QuickDASH or QuickDASH-9, in the BCS population.

The present study included 216 BCS, and the same sample was used for all analyses. Future studies should include a more significant BCS sample, allowing for segmenting data for an MLE and CFA analysis. A larger sample will provide a more robust and demanding data analysis.

As for strengths, as far as we know, the present research has demonstrated the validity of the content and construct of the ULFI-Sp in the BCS. Furthermore, this is the first version of the ULFI-Sp abbreviated to 14 items that maintain the original psychometric properties. Finally, factor analysis by CFA was tested in the analyses.

## 5. Conclusions

This study analyzed the psychometric properties of the ULFI-Sp in a sample of the BCS, resulting in a new ULFI-Sp short version. In light of the present results, the developed version of the ULFI-SP is preferable to assess the upper limb function in Spanish women after breast cancer surgery. Given the high prevalence of ULD in this population and the wider versions of ULFI across different languages, this study’s results may be transferred to clinical practice and integrated as part of upper limb assessment after breast cancer.

## Figures and Tables

**Table 1:** Participant descriptive and clinical variables.

	Mean (SD)	Min–Max
Age (years)	51.64 (9.10)	32.0–69.0
BMI (Kg/m^2^)	27.97 (5.39)	17.60–43.50
Years from diagnosis	2.43 (2.16)	0–13
Surgical intervention		Percentage (n)
	Lumpectomy	11% (23)
	Lumpectomy and sentinel lymph node	38% (83)
	Lumpectomy and axillary dissection	24% (52)
	Mastectomy	27% (58)
Cancer treatment		
	Chemotherapy	88.5% (191)
	Radiotherapy	90.6% (195)
	Hormone therapy	82.2% (190)
	Monoclonal antibody	29.3% (63)
Current treatment		
	None	23% (49)
	Radiotherapy	6% (13)
	Monoclonal antibody	6% (13)
	Hormone therapy	65% (140)

**Table 2:** Twenty-five-item average score, correlation and communalities from ULFI-Sp in BCS and fourteen-item short version communalities.

Item Number	Item Description	Item Average Score (SD)	Inter-Item Correlation	Communalities	
				25 Item (Original Version)	14 Item (Short Version)
1	Stays at home most of the time	0.18 (0.34)	0.516	0.247	
2	Changes positions frequently	0.38 (0.43)	0.621	0.357	0.483
3	Avoids heavy jobs	0.59 (0.45)	0.541	0.271	
4	Rests more often	0.52 (0.45)	0.647	0.388	0.375
5	Gets others to do things	0.29 (0.37)	0.506	0.237	
6	Pain is constant	0.26 (0.38)	0.702	0.456	0.535
7	Lifting and carrying	0.47 (0.44)	0.703	0.458	0.471
8	Appetite affected	0.18 (0.35)	0.399	0.147	
9	Walking/normal recreation/sport	0.35 (0.41)	0.584	0.316	0.321
10	Home/family duties and chores	0.33 (0.40)	0.771	0.551	0.733
11	Sleeps less well	0.46 (0.45)	0.480	0.214	
12	Assistance with personal care and hygiene	0.02 (0.14)	0.352	0.115	
13	Regular daily activities, work/social	0.31 (0.41)	0.659	0.402	0.391
14	More irritable/bad tempered	0.29 (0.40)	0.482	0.215	
15	Feeling weaker or stiffer	0.38 (0.42)	0.729	0.493	0.553
16	Transport independence	0.08 (0.22)	0.398	0.147	
17	Arm in shirt sleeve/dressing	0.15 (0.30)	0.531	0.261	
18	Writing/using keyboard or mouse	0.06 (0.21)	0.491	0.223	
19	Doing things at/above shoulder height	0.22 (0.34)	0.618	0.354	0.486
20	Eating and using utensils	0.05 (0.18)	0.434	0.175	
21	Holding or moving dense objects	0.21 (0.36)	0.657	0.400	0.408
22	Drops things, causing minor accidents	0.16 (0.32)	0.572	0.303	0.299
23	Uses other arm more often	0.48 (0.46)	0.588	0.321	0.323
24	Difficult button key coins taps	0.23 (0.37)	0.629	0.357	0.337
25	Difficult opening, holding, pushing or pressing	0.42 (0.42)	0.698	0.452	0.468

**Table 3:** Factor structure and factor analysis from ULFI 25-items original version and 14-items short version.

Psychometric Properties	ULFI	25-Item Model (Original Version)	14-Item Model (Short Version)
AFE	KMO	0.889	0.920
	Barlett’s test		
	Chi-squared	2087.167	1171.591
	Df; *p*	300; <0.000	91; <0.000
CFA	CFI	0.802	0.905
	RMSEA	0.075	0.069

## Data Availability

The corresponding author will provide data upon reasonable request.
